# Successful Laparoscopic Treatment of Morgagni’s Hernia in an Elderly Female Presenting as a Hypoxemic Hypercapnic Respiratory Distress

**DOI:** 10.7759/cureus.54876

**Published:** 2024-02-25

**Authors:** Farah Assi, Ali Mecheik, Hassan Zghaib, Houssein Haidar Ahmad

**Affiliations:** 1 Infectious Diseases, Internal Medicine, Lebanese University Faculty of Medicine, Beirut, LBN; 2 Internal Medicine, Intensive Care, Saint George Hospital, Beirut, LBN; 3 Intensive Care, Saint George Hospital, Beirut, LBN; 4 Anaesthesiology, Saint George Hospital, Beirut, LBN; 5 General Surgery, Saint George Hospital, Beirut, LBN; 6 General Surgery, Lebanese University, Beirut, LBN

**Keywords:** right-to-left shunt, pulmonary atelectasis, hypoxia and hypercapnia, minimally invasive laparoscopy, adult congenital diaphragmatic hernia, morgagni’s hernia

## Abstract

Morgagni's hernia (MH) occurs when the abdominal viscera herniates into the thoracic cavity through a congenital anatomical defect in the diaphragm, termed the foramen of Morgagni. Although it is more frequently detected in childhood, its delayed presentation in adults and the elderly could be easily overlooked due to the non-specificity of its symptoms. Here, we report the case of an elderly female who presented purely with dyspnea and desaturation, necessitating admission to the intensive care unit. Her computed tomography (CT) scan revealed the presence of MH with complete lobar collapse. Laparoscopy was successful in reducing the hernia, and the patient improved with a good prognosis. Surgical treatment for MH is advised for all cases in order to prevent the occurrence of serious complications.

## Introduction

The diaphragmatic hernia of Morgagni was first described in 1769 by Morgagni, as an anterior, retrosternal, or parasternal muscular defect in the diaphragm, through which herniation of the abdominal viscera into the thorax occurs [1]. The occurrence of a congenital diaphragmatic hernia is not uncommon, as it occurred in one in 3,156 births according to a population-based epidemiological study in 2017 [2]. Morgagni's hernia (MH) constitutes 1-5% of congenital diaphragmatic hernias, and although many are discovered incidentally, others may present with obstructive symptoms, both of which surgical treatment is advised to prevent complications [3-4]. Here, we report a case of MH occurring in an elderly female presenting purely with respiratory symptoms.

## Case presentation

A 75-year-old female presented to the emergency room complaining of new-onset progressive dyspnea at rest accompanied by right-sided chest discomfort, which started 10 days prior to presentation. She denied any other symptoms. She was a previous smoker and had no past medical history, and her surgical history was only significant for left hip replacement two years ago. Since the start of her dyspnoea, she was unable to perform basic daily life activities. She weighed 80 kg, and her body mass index (BMI) was 33.3 kg/m².

**Figure 1:**
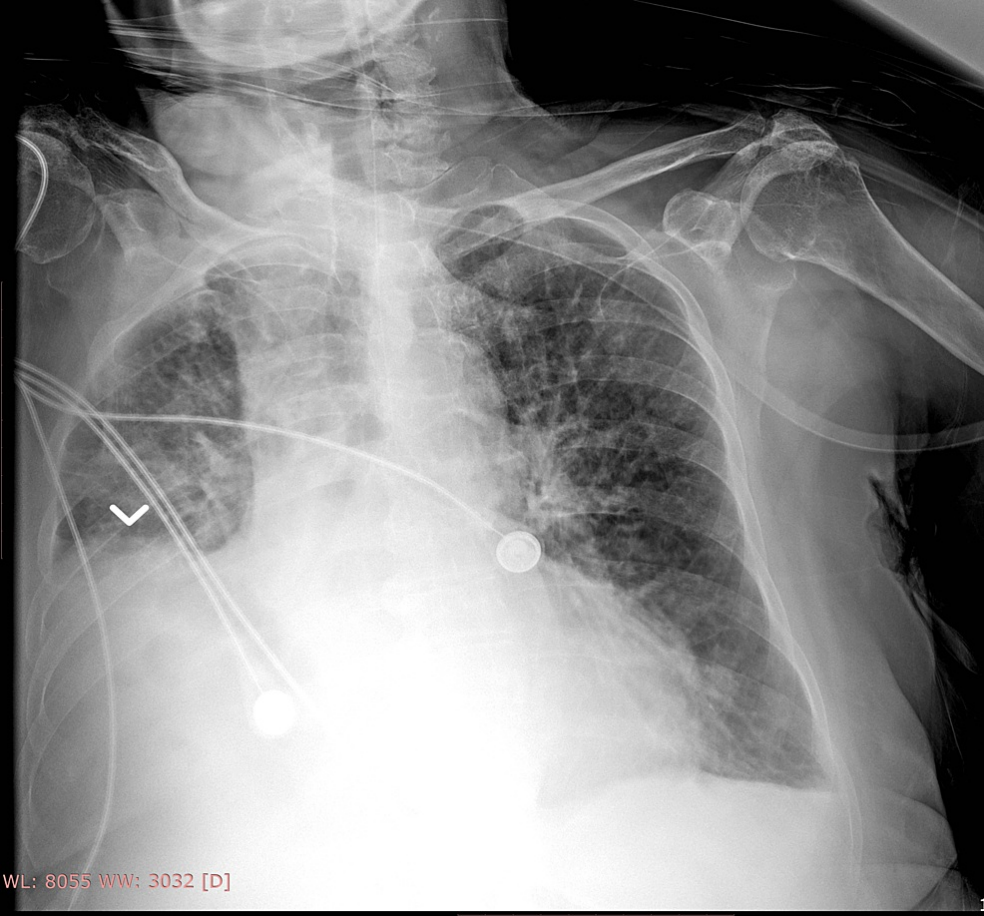
Portable chest X-ray at admission showing right lung lower lobe collapse (white arrowhead)

Upon presentation, the patient appeared ill. She was hypertensive (blood pressure 190/92 mmHg), tachypneic, desaturated (89%) on room air, afebrile, and slightly drowsy. Physical examination only revealed decreased air entry on the right side from mid to low lung auscultation fields. Abdominal examination was normal. Arterial blood gases showed hypoxemic respiratory acidosis with CO₂ retention (pH 7.25, PCO₂ 85 mmHg, PO₂ 66 mmHg, and HCO₃ 37 mEq/l). Bilevel positive airway pressure (BiPAP) was applied with 10 L/min oxygen (I-PAP = 14, E-PAP = 6). Sublingual captopril (50 mg) was given for hypertension. A basic metabolic panel revealed only a slightly elevated C-reactive protein level (CRP) (1.7 mg/dl, normal range 0-1 mg/dl). Portable chest X-ray demonstrated right lung lower lobe collapse (Figure 1).

**Figure 2:**
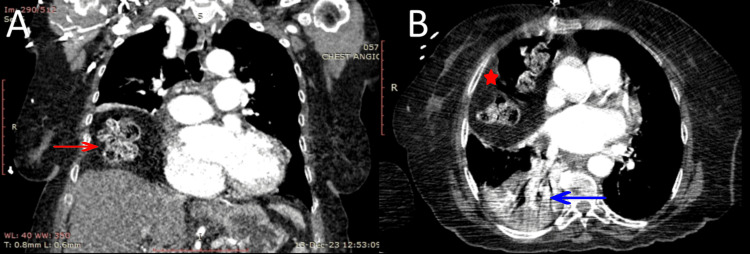
Angiogram scan of the chest showing the presence of bowels (A, red arrow; B, red star) in the right thoracic cavity with complete collapse of the right lower lobe and mild partial obstruction of the right lower bronchus (B, blue arrow). A, coronal view; B, transverse view.

The patient was admitted to the intensive care unit (ICU). She was suspected of having a pulmonary embolism (PE), so an angiogram scan of the chest was done. It ruled out the presence of a PE, but it showed right-sided herniation of the transverse colon into the thorax, without strangulation, causing a total collapse of the right lower lobe with partial obstruction of the right lower bronchus (Figure 2).

The patient was diagnosed with right-sided MH causing right lower lobe complete atelectasis. As a consequence, perfusion of the airless collapsed alveoli resulted in deoxygenated blood bypassing the lungs into the systemic circulation, thus producing a right-to-left shunt effect. She remained dependent on BiPAP with high oxygen requirements until surgery.

**Figure 3:**
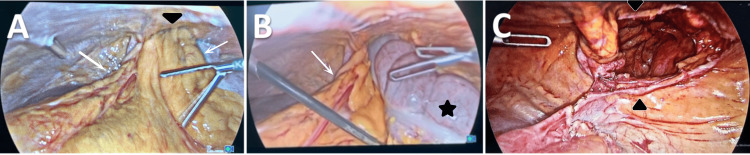
Intraoperative images. A and B: Morgagni’s hernia containing the omentum (white arrows) and the transverse colon (black star) seen herniating through the diaphragmatic defect (black arrowheads); C: the diaphragmatic defect (black arrowheads) sized (5 x 8) cm after the reduction of the hernia.

Laparoscopic repair of MH was performed under general anesthesia. The patient was placed supine and then positioned in the reverse Trendelenburg position. The abdomen was accessed using the open technique at the umbilicus. CO₂ insufflation was achieved at a pressure of 12 mmHg. Three trocars were placed. Exploration revealed herniation of the transverse colon and the greater omentum through the foramen of Morgagni (Figure 3A, 3B). The herniated contents were reduced, and the hernia sac was completely dissected and excised. The hernia defect size was (5 x 8) cm (Figure 3C).

**Figure 4:**
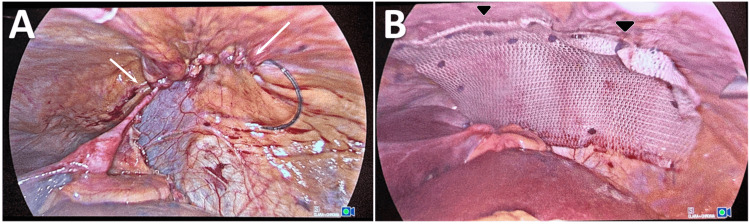
A: Closure of the defect (white arrows); B: use of a 15 x 10 cm dual mesh to reinforce the closure (black arrowheads).

The foramen was closed using a running 3/0 v-lock suture (Figure 4A). A (10 x 15) cm Paritex™ optimized composite dual mesh was applied to reinforce the closure, which was secured to the diaphragm using a 5 mm AbsorbaTack™ fixation device and interrupted 3/0 polypropylene sutures (Figure 4B). The total operative time was 60 minutes. The patient tolerated the procedure well and then was transferred postoperatively to the ICU.

**Figure 5:**
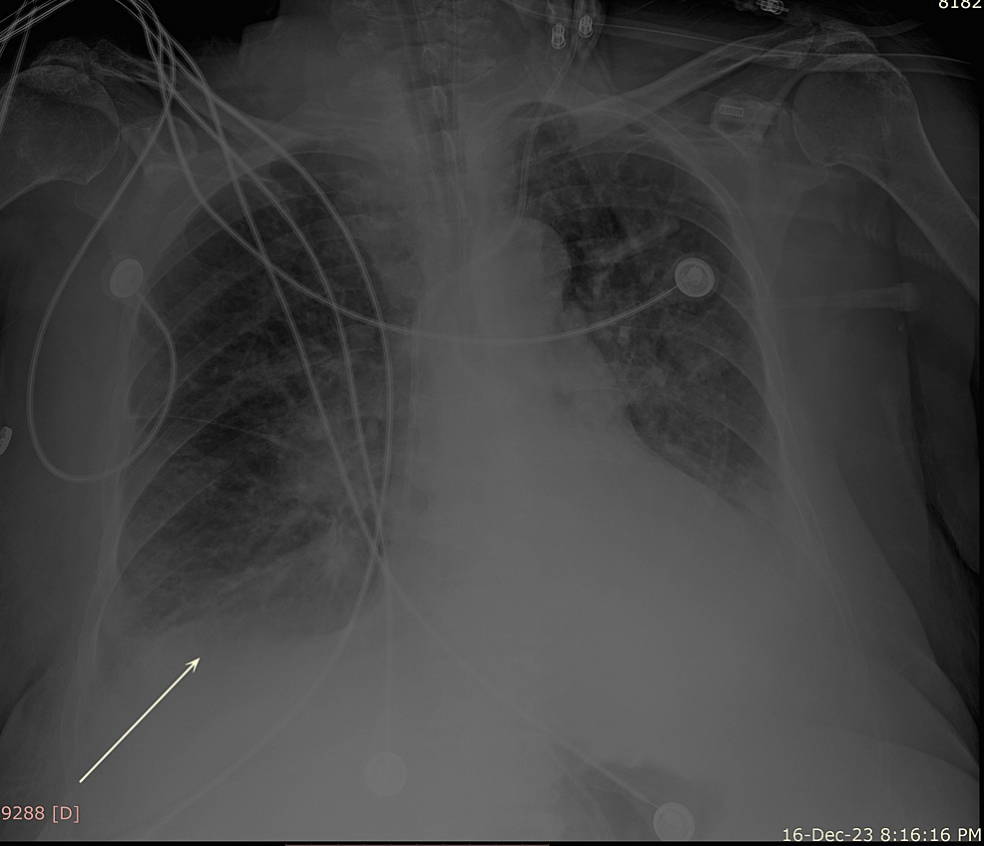
Postoperative portable chest X-ray showing resolution of the right lower lobe collapse and re-expansion of the lung (white arrow).

After the correction of the patient’s hernia, the patient’s right lower lung lobe re-expanded (Figure 5).

Her hypoxemia and hypercapnia were resolved, and she was liberated from BiPAP. Her course in the hospital was complicated by the occurrence of slow-rate atrial fibrillation. She was discharged home on room air after a total number of 23 in-hospital days.

## Discussion

MH is one of the four described congenital diaphragmatic hernias, resulting from an anatomical defect in the sternocostal trigone, termed the foramen of Morgagni [4]. Due to the relative paucity and non-specificity of symptoms, many are not diagnosed until adulthood, making it difficult to explore the overall prevalence in the adult population [5-6]. Symptomatic MHs in adults present more frequently in females in their fifth decade of life [7]. Changes in the intraabdominal pressure related to pregnancy, obesity, constipation, trauma, and cough have been linked to the development of these hernias, and the ensuing vascular compromise may result in transient or complete ischemia to the affected organs [8]. MHs are usually right-sided, owing to the presence of the heart and the pericardial sac on the left side [6]. Consequently, the occurrence of MH on the left side is less frequent, whereas bilateral MH is rarely reported [7,9].

The percentage of asymptomatic patients varied across the literature, and it ranged from a reported 10.3% [7] to 28% [9] and 32.3% [10]. The presenting complaints were for the most part gastrointestinal (GI) and respiratory [10-11]. Abdominal pain, nausea and vomiting, dysphagia, reflux disease, and bowel obstruction were the most commonly described GI complaints [7,9,11], whereas respiratory symptoms consisted mainly of dyspnea, cough, and chest pain [9,11-12]. Both the contents and the size of the hernia play a major role in the symptomatology, which can be summed as follows: herniation of the small or large bowel can present with intestinal obstruction, herniation of the omentum produces pain, that of the stomach may induce postprandial abdominal discomfort or reflux disease, whereas the size of the hernia has been linked to the development of respiratory symptoms [6,11]. The most frequently herniated contents are the omentum and the transverse colon, followed by the stomach, liver, and small intestine [7,10,13].

A computed tomography (CT) scan is considered the gold standard for diagnosis, being accurate, and able to provide information regarding the presence of a concomitant strangulation requiring emergency management [14]. MHs can be easily missed on chest radiographs depending on the contents and the size of the hernia [15]. Magnetic resonance imaging provides accurate diagnosis as well [6]. Other used modalities are contrast enemas, upper GI studies, and upper GI endoscopies [9].

Due to the risk of strangulation, surgery should be performed for all cases diagnosed with MH, regardless of the presence or absence of symptoms [10]. Several surgical methods have been performed: open abdominal approach (laparotomy), open thoracic approach (median sternotomy or thoracotomy), laparoscopy, and thoracoscopy [8]. No clear consensus is provided regarding the choice of optimal surgical procedure [6]. The open abdominal approach is the most used in emergent and complicated cases and allows for easy reduction of the hernia contents, screening for other defects, and repair of intra-abdominal pathologies [9]. Thoracotomy has been widely used with good results and allows for defect repair and pleural and pericardial sac adhesion clearance [16]. The growing use of laparoscopy is reported to be safe, effective, and ideal for providing enhanced visualization and manipulation of the hernia sac [17]. While laparoscopy has been associated with a shorter time to recovery, lower postoperative complication rate, and cosmetic benefit, it may, however, prove to be suboptimal in complicated and emergent cases [8].

The decision to excise the hernia sac remains debated, as its removal can damage the surrounding anatomical structures [14]. However, higher rates of seromas, hematomas, and hernia recurrence have been reported after retaining the sac [11]. The recommendation for the use of mesh to reinforce the closure is weak. In defects larger than 3 cm, the use of a mesh assists in reducing the resulting tissue tension [1].

Our patient was an elderly female, with class 1 obesity as a risk factor, who presented for new dyspnea and was found to be in hypoxemic hypercapnic respiratory acidosis. The diagnosis of MH was established by a CT scan of the chest. The hernia caused complete collapse of the right lower lobe, producing a right-to-left shunt effect. Her dyspnea and desaturation were not otherwise explained. She underwent laparoscopy for the reduction of the hernia and sac excision. Because the defect was large (5 x 8 cm), a mesh was used to fortify the closure. Expansion of the collapsed lobe was achieved after the operation. She required a relatively prolonged hospital stay until her respiratory status improved and she no longer needed oxygen supplementation. No postoperative complications were seen.

## Conclusions

MH can be completely asymptomatic or can be associated with abdominal or respiratory symptoms, depending on the qualities of the hernia. Obstruction and strangulation are severe consequences. Respiratory symptoms are related to the compressive effects the herniated viscera exert on the lung. Surgical treatment is recommended for all cases. Laparoscopy is safe and offers successful results with low morbidity, shortened hospital stay, and fewer complications. Complicated hernias are better operated via the open abdominal approach. The excision of the hernia sac and the use of a mesh are still debated among authors.
